# Pharmacokinetics and Pharmacodynamics of Analgesic and Anesthetic Drugs in Patients During Cardiac Surgery With Cardiopulmonary Bypass: A Narrative Review

**DOI:** 10.1213/ANE.0000000000007564

**Published:** 2025-05-16

**Authors:** Anne Beukers, Jennifer Breel, Charissa van den Brom, Aryen Saatpoor, Jolanda Kluin, Douglas Eleveld, Markus Hollmann, Henning Hermanns, Susanne Eberl

**Affiliations:** From the 1 Department of Anesthesiology, Amsterdam UMC, Amsterdam, the Netherlands; 2 Department of Intensive Care Medicine, Amsterdam UMC, Amsterdam, the Netherlands; 3 Faculty of Medicine, University of Amsterdam, Amsterdam, the Netherlands; 4 Department of Cardiothoracic Surgery, Erasmus MC, Erasmus University Rotterdam, Rotterdam, the Netherlands; 5 Department of Anesthesiology, UMC Groningen, Rijksuniversiteit Groningen, Groningen, the Netherlands.

## Abstract

Cardiopulmonary bypass (CPB) impacts pharmacokinetics and -dynamics of drugs used during cardiac surgery. These alterations can lead to changes in drug efficacy resulting in under- or overdosing. This review summarizes current knowledge on the effects of CPB on commonly used intraoperative and continuously administered anesthetics and analgesics. Out of 197 articles initially identified, 22 were included in the final review. The breakdown of studies by main topic was as follows: propofol (9 articles), sevoflurane (4), remifentanil (3), isoflurane (2), fentanyl (2), and sufentanil (2), and alfentanil (1). The initiation of CPB typically results in hemodilution and hypothermia, leading to a decrease in total plasma concentration combined with an increase in unbound plasma concentrations. This phenomenon has varying implications for different drugs: For propofol and sevoflurane, lower doses may be required during CPB to achieve the same anesthetic effect. Fentanyl and sufentanil plasma concentrations decrease by 25% on average at CPB initiation due to an increased volume of distribution, followed by an increase during CPB, with sufentanil, showing an almost 50% increase post-CPB. This implies that an additional bolus before CPB initiation should be considered, followed by a reduction of the maintenance dose to prevent prolonged sedation. Remifentanil plasma concentration decreases at CPB initiation, which implies that higher initial- or adjusted maintenance dose should be considered in normothermic patients. However, under hypothermic conditions, infusion rates should be decreased by 30% for every 5°C decrease in temperature. Alfentanils, total plasma concentration decreases during CPB, while its free fraction remains unaltered, indicating that no further adjustments are necessary. Target-controlled infusion (TCI) models for propofol (Schnider, Marsh, and PGIMER [Postgraduate Institute of Medical Education and Research]) and remifentanil (Minto) were found to be inaccurate in the context of CPB. Based on the included studies, the use of these pharmacokinetic models is not recommended. In conclusion, dosing inaccuracies resulting in adverse events in on-pump cardiac surgery underscore the importance of understanding the pharmacokinetics and -dynamics of anesthetic and analgesic drugs during CPB. The clinical implication of the altered drug responses after CPB remains challenging in this high-risk population. Key takeaways include the necessity of considering patient-specific factors, utilizing objective monitoring tools, and recognizing potential drug alterations due to CPB.

**See Article, page 1**

Cardiopulmonary bypass (CPB) is an essential component of cardiac surgery maintaining circulation, oxygenation, decarboxylation, and temperature management despite cardiac arrest. While it has long been recognized that CPB alters the pharmacokinetics and pharmacodynamics of anesthetic drugs,^1,2^ the clinical implications of these alterations remain challenging to translate into precise, personalized drug dosing guidelines.

Previous reviews have highlighted significant changes in drug behavior during CPB,^3^ including alterations in distribution, elimination, and plasma concentrations.^4^ Key factors contributing to these changes include hemodilution, an increased volume of distribution at CPB initiation, potential adhesion to CPB tubing, or altered plasma protein binding, and redistribution during CPB, all of which may result in an initial decrease plasma concentration of the drug, followed by an increase. However, the evolution of CPB techniques, such as smaller priming volumes, mini-extracorporeal circulation systems, and also retrograde autologous priming mitigated some of these effects.^5,6^ Despite these advancements, clear guidance on optimizing drug dosing during CPB remains elusive.

Anesthesia providers face the complex task of maintaining efficient control of anesthesia depth while ensuring stable hemodynamics, short awakening times, and rapid extubation. Cardiac surgery, in particular, is recognized as high-risk for accidental awareness,^7^ with a reported incidence of 0.012% to 0.5%,^5,8^ which is higher compared to general anesthesia (0.017%).^5,9^ Factors during CPB contribute to underdosing of anesthetic drugs, increasing the risk of awareness. Conversely, increased anesthetic depth is associated with adverse outcomes,^10,11^ most notably postoperative delirium, which is linked to postoperative complications and prolonged hospital stays.^12^ However, there is a gap in our understanding of the optimal plasma and effect-site concentrations of analgesics and anesthetics during on-pump cardiac surgery. This knowledge deficit impacts our ability to simultaneously prevent intraoperative awareness, promote faster postoperative awakening, maintain hemodynamic stability, and reduce or eliminate the need for vasoconstrictive or inotropic support.

This review summarizes current knowledge on the effects of CPB on anesthetic drug behavior, with a focus on the most commonly used intraoperative and continuously administered anesthetics and analgesics. We also explored the impact of modern CPB techniques, the role of target-controlled infusion (TCI) models, and the influence of patient factors such as sex on drug pharmacokinetics and pharmacodynamics.

By critically evaluating the available evidence, this review seeks to provide anesthesiologists with practical insights for managing anesthesia during CPB, while also identifying key areas where further research is needed to enhance patient safety and improve outcomes in cardiac surgery.

## METHODS

We searched the MEDLINE (PubMed) and Embase database for English-, Dutch- or German-language publications from January 2000, through January 2022, with an updated search on January 8, 2025, related to the pharmacokinetics and -dynamics of the most commonly used intraoperative continuously administered anesthetics and analgesics during cardiac surgery using CPB. Elements of the search are included in the Supplemental Digital Content, Table 1, http://links.lww.com/AA/F313. Articles published before 2000 were excluded, since the changes in plasma drug concentrations in studies up to 2000 are well summarized.^3^ Only observational-, clinical-, and randomized studies were included. Relevant references cited by identified articles were included.

Articles were selected based on commonly used and continuously administered analgesics (fentanyl, sufentanil, remifentanil, alfentanil, morphine, ketamine) and anesthetics (sevoflurane, isoflurane, propofol, midazolam). Less commonly or not continuously administered drugs were excluded from review including analgesics such as ketorolac, hydromorphone, meperidine, and oxycodone as well as anesthetics like desflurane and etomidate.

The records were entered into a database (Rayyan Qatar Computing Research Institute). Screening was independently performed by 3 reviewers (A.S., S.E., and J.B.), with an updated screening by 2 (A.B. and S.E.). Initial screening was based on title and abstract, followed by full-text screening of the eligible articles for final inclusion. Duplicates were identified and removed manually. Of 197 articles, 22 articles were included, including studies with propofol as main topic in 9 articles,^13–21^ sevoflurane in 4,^22–25^ remifentanil in 3,^26–28^ isoflurane in 2,^25,29^ fentanyl,^30,31^ and sufentanil in 2,^32,33^ and alfentanil in 1 article.^34^

**Table 1.** Influence of Different TCI Models on Drug Concentration During CPB

Author, year	Study design and population	N	Anesthetic drug and model	Main findings and clinical implication
Lee et al.,^18^ 2018	Observational pilot, CABG, CABG/valve	10	Propofol, Schnider	Under-prediction of plasma concentration. Clinical implication: Risk of overdosing with Schnider model during CPB.
Mathew et al.,^13^ 2016	Randomized, CABG, valve, coronary heart disease	23	Propofol, Marsh and PGIMER	Overprediction of plasma concentration. Clinical implication: Risk of underdosing Marsh and PGIMER model during CPB.
Cho et al.,^26^ 2017	Randomized, CABG	56	Remifentanil, Minto	Under-prediction of plasma concentration in moderate-deep hypothermia. Clinical implication: Risk of overdosing with Minto model during CPB.
Scherrer et al.,^28^ 2022	Observational, CABG, aortic valve, CABG/aortic valve	58	Remifentanil, Minto	Overprediction of plasma concentration in prebypass, and postbypass period. Accurate prediction of plasma concentration during normothermic CPB. Clinical implication: risk of underdosing in pre- and post-CPB period. Good accuracy during CPB.

Abbreviations: CABG, coronary artery bypass graft; CPB, cardiopulmonary bypass; PGIMER, Postgraduate Institute of Medical Education and Research.

**Table 2.** Influence on Pharmacodynamics in Patients Undergoing Cardiac Surgery With CPB

Author, year	Study design and population	N	Anesthetic drug	Main findings and clinical implication
Inoue et al.,^20^ 2001	Nonrandomized, CABG	21	Propofol	Propofol infusion does not particularly potentiate an increase in free fatty acids compared to isoflurane and midazolam. Clinical implication: elevated free fatty acids can increase the severity of myocardial ischemic damage. Propofol does not increase free fatty acid levels.
Reinsfelt et al.,^21^ 2011	Observational, CABG, valve, CABG/valve	16	Sevoflurane	Sevoflurane in burst-suppression dose (inspired concentration 3.4%) decreases cerebral oxygen extraction more than cerebral blood flow velocity. Clinical implication: sevoflurane has direct intrinsic cerebral vasodilatory effect inducing partial loss of flow-metabolism coupling.
Reinsfelt et al.,^29^ 2003	Observational, CABG, valve, CABG/valve	16	Isoflurane	Isoflurane in burst-suppression dose decreases cerebral blood flow velocity more, than cerebral oxygen extraction. Compared to opioid-based anesthesia CPP relatively decreased by 70% with isoflurane. Clinical implication: isoflurane has a direct vasodilating effect, in addition to its effect on cerebral metabolism, disrupting metabolic autoregulation.

Abbreviations: CABG, coronary artery bypass graft; CPP, cerebral perfusion pressure.

**Table 3.** Adverse Events and PK/PD Alterations After CPB: Impact and Future Directions

Adverse event	Frequency	Contribution of PK/PD	Currently used mitigation strategies	Knowledge gap	Proposed focus for future research
Awareness	~ 0.012%–0.5%^5,8^	Inadequate anesthetic levels due to altered distribution at CPB initiation (eg, dilution drug binding proteins, increased volume of distribution).	Adjust dose based on altered plasma concentrations during CPB, utilize BIS/EEG monitoring when available.	Insufficient validation of TCI models for CPB setting, understanding of coadministration of drugs, effect of patient-specific factors.	Development of PK/PD models, including coadministration of drugs, larger sex-balanced, weight-matched study population.
Profound hypotension	High interindividual variability, reported up to	Diminished plasma protein binding with increased free fractions, redistribution during CPB.	Limit excessive dosing of propofol and sevoflurane, utilize BIS/EEG monitoring when available.	Lack of personalized drug dosing strategies to prevent overdosing.	Studies to quantify the impact altered PKPD of anesthetics due to CPB on hypotension and its associated complications.
Delayed awakening	Unknown, poorly quantified.	Drug accumulation due to altered clearance as a consequence of hypothermia.	Limit excessive dosing of propofol, sufentanil, and fentanyl, utilize BIS/EEG monitoring when available.	Redistribution and clearance after CPB, and its effect on delayed awakening.	Studies on optimizing clearance after CPB, and in the postoperative period.
Variability in drug response	High interindividual variability	Differences in plasma protein, volume of distribution, and metabolism rates during CPB.	Adjustment of drug doses based on weight.	Limited understanding of how individual variability impact drug behavior during CPB.	Cohort studies with large sex-, and weight-matched groups to explore the individual variability. Further customization of priming and perfusion.
Adverse long-term effects	Variability based on adverse outcome	Persistent under/overdosing during CPB potentially contributes to complications like organ dysfunction or neurocognitive dysfunction.	Postoperative monitoring for organ dysfunction.	Poorly quantified postoperative outcomes of PK/PD alterations after CPB.	Long-term studies to assess impact of CPB-related PK/PD alterations on recovery, and morbidity.

Abbreviations: BIS, bispectral index; CPB, cardiopulmonary bypass; EEG, electroencephalographic; PK/PD, pharmacokinetics and pharmacodynamics; TCI, targeted controlled infusion.

A large part of the articles, related to propofol and remifentanil, examined the effect of different TCI models.^13,18,26,28^ This trend warranted the creation of a dedicated subsection TCI (Table 1). TCI systems use pharmacokinetic/dynamic drug models to calculate and administer drug infusion, aiming to achieve and maintain target concentration in plasma or at effect sites. The system’s effectiveness depends on the accuracy of these models, which reflects drug uptake and distribution in the body’s tissues. These models typically use 2- or 3 abstract compartments to represent tissues with similar pharmacokinetic properties.^35^ To enhance accuracy, models often incorporate patient-specific factors such as age, weight, height, and sex. For example, the Schnider propofol and the Minto remifentanil model both account for sex indirectly through its influence on lean body mass calculation, which influence predicted drug clearance and distribution.^28,36,37^

## RESULTS

### Influence of CPB on Pharmacokinetics and -Dynamics

The after sections provide a detailed description of the main findings of the pharmacokinetics and -dynamics of reviewed drugs in patients undergoing on-pump cardiac surgery. The characteristics of included studies and main findings on the influence of CPB on the pharmacokinetics of the reviewed drugs are summarized in Supplemental Digital Content, Table 2, http://links.lww.com/AA/F313. Details of included studies and main findings on the accuracy of different TCI models in Table 1, characteristics of included studies and main findings on the pharmacodynamics in Table 2, and pharmacokinetic and -dynamic drug alterations during CPB in relation to adverse events with knowledge gaps and future directions are summarized in Table 3.

### Pharmacokinetics and Pharmacodynamics of Anesthetics

#### Propofol

Nine articles focused on propofol as the main topic.^13–21^ Propofol pharmacokinetics and pharmacodynamics are significantly altered during CPB. On initiation of CPB, the plasma concentration of propofol decreases as a result of hemodilution and an increased volume of distribution, which in turn increases the unbound faction of propofol, 2- to 5-fold, mainly due to lower plasma albumin levels.^16,21^ After termination of CPB, plasma concentration increases again. This effect has been linked to hypothermia-induced slowing of propofol metabolism, a shortened biological half-life or increased total plasma clearance during and due to CPB.^13,14^

Taken together, these effects suggest an increase in the unbound fraction of propofol in plasma, contributing to more intense hemodynamic and central nervous system depressant effects during CPB, also resulting in longer awakening times and delayed extubation in on-pump versus off-pump procedures.^14,15^ Closely related, patients with higher propofol dosages during CPB (6 mg kg^−1^ h^−1^) exhibited more frequent burst suppression rates (BSR) compared with those receiving lower dosages (4 mg kg^−1^ h^−1^). The propofol concentration in patients receiving higher dosages (6 mg kg^−1^ h^−1^) was 1.5 times higher than in those receiving lower dosages (4 mg kg^−1^ h^−1^), proportional to the dose. While the unbound concentration increased 2-fold in both groups, BSR significantly increased after CPB initiation in patients receiving the higher dose, whereas it gradually increased in patients receiving the lower dose. Although BSR showed only a moderate correlation with the concentration of unbound propofol, it may nevertheless indicate that the change in the effective plasma concentration of propofol has a significant cerebral effect.^16^ This also matches the results of Yoshitani et al,^17^ who reported that BSR time gradually increased during CPB in patients receiving higher propofol dosages (5 or 6 mg kg^−1^ h^−1^) in contrast to patients receiving 4 mg kg^−1^ h^−1^ (Supplemental Digital Content, Table 2, http://links.lww.com/AA/F313).^17^ These results indicate that continuing pre-CPB propofol dosing during hypothermic CPB may lead to too high effective plasma levels with consecutive increased hemodynamic- and cerebral side effects and that 4 mg kg^-1^ h^-1^ may be sufficient.

### TCI Models

Two studies used TCI-measured plasma propofol concentration at specific time points before, during, and after CPB to evaluate the bias and accuracy of the TCI system.^13,18^ Findings on the performance of propofol TCI among patients undergoing on-pump cardiac surgery are inconsistent due to the use of different pharmacokinetic models.^13,18^ Importantly, no pharmacokinetic model could predict the exact plasma concentration of propofol during CPB. While the Schnider pharmacokinetic model tended to under-predict plasma concentration during CPB, especially in underweight patients,^18^ the Marsh and PGIMER showed overprediction.^13^ In obese patients (82% female) age, but not sex or obesity, did influence the pharmacokinetics of propofol during hypothermic CPB.^19^ The lack of influence of obesity could be attributed to reduced tissue perfusion during CPB and a smaller volume of distribution.^19^

### Volatile Anesthetics: Sevoflurane and Isoflurane

Two studies examined sevoflurane plasma concentration during CPB.^23,24^ Both studies, using gas chromatography, observed a decrease in sevoflurane plasma concentration measured at initiation of CPB, followed by a gradual increase and decrease at the end with a stable bispectral index (BIS).^23,24^ Contributing factors of the decrease at the start of the CPB may have been a decrease in hematocrit and body temperature since both affect blood/gas solubility coefficient of sevoflurane.^23,24^

The gradual increase during CPB may be caused by an increased tissue capacity for volatile anesthetics due to hypothermia and accumulation of anesthetics by the oxygenator and CPB circuit. Moreover, alterations in blood flow distribution and hypotension, resulting in a reduced drug metabolism and elimination may be caused by this mechanism.^23,24^ These results suggest that end-tidal sevoflurane reflects sevoflurane plasma concentration well and therefore adequate, safe anesthesia can be maintained during CPB with a lower anesthetic requirement. One study examined the wash-in, and washout kinetics of sevoflurane and isoflurane from CPB initiation until weaning with mini-CPB systems.^25^ Wash-in and washout kinetics for sevoflurane and isoflurane were comparable, despite the difference in relative blood-gas solubility, from which a faster wash-in, and washout kinetics were expected with sevoflurane.^25^ However, during CPB, a higher dose of sevoflurane was required compared to isoflurane to achieve the same depth of anesthesia (BIS 30–45).^25^

Reinsfelt et al studied the effects of sevoflurane^22^ and isoflurane^29^ on cerebral pressure-flow autoregulation and flow-metabolism coupling during CPB. Cerebral pressure-flow autoregulation is a sensitive mechanism that minimizes alterations in cerebral blood flow (CBF) when cerebral perfusion pressure changes. When sevoflurane and isoflurane were used in concentrations that induced BSR in electroencephalography (4–6, and 6–9 bursts per minute for sevoflurane and isoflurane, respectively), CBF velocity and cerebral oxygen extraction decreased with both volatile anesthetics (17% and 23%, respectively, for sevoflurane, and 27% and 13% for isoflurane).^22,29^ This finding suggests that sevoflurane and isoflurane both have a direct intrinsic cerebral vasodilatory effect, in addition to its effect on cerebral metabolism, that induces a partial loss of flow-metabolism coupling (Table 2). This effect seems more pronounced with isoflurane. A certain decrease in cerebral perfusion pressure resulted in a 30% more pronounced absolute decrease, and a 70% relative decrease, in CBF velocity in isoflurane compared with opioid-based anesthesia.^22^

Clinicians using sevoflurane or isoflurane during CPB should monitor end-tidal concentrations, expect lower anesthetic requirements during CPB (more pronounced with isoflurane than with sevoflurane), and be aware of its potential effects on CBF and metabolism.

### Pharmacokinetics and Pharmacodynamics of Analgesics

#### Fentanyl

Pharmacokinetics of fentanyl show fluctuations in plasma concentration averaging 25% at the onset and separation from CPB.^31^ This transient phenomenon may be explained by the peripheral distribution and later redistribution of fentanyl as a lipophilic agent to fat tissue and the pulmonary first-pass metabolism after restoration of pulmonary blood flow.^38^ This process helps to moderate the immediate increase and decrease of fentanyl concentration, preventing significant fluctuations (Supplemental Digital Content, Table 2, http://links.lww.com/AA/F313).^31^ Although these fluctuations are temporary, they can be clinically relevant when extubation is planned at the end of a short operation. Administering an additional bolus of fentanyl before the initiation of CPB may help reduce the initial drop in plasma concentration. However, this could lead to an increased free fraction of fentanyl due to hemodilution, potentially worsening the rise in plasma concentration after weaning. Therefore, given the redistribution and the resulting gradual increase in plasma concentration during CPB, anesthesiologists should consider reducing the dose if an additional bolus is given.

Two studies investigated the effectiveness of a 3-^31^ or 2- and 3-compartment^30^ pharmacokinetic model for predicting fentanyl levels during CPB by using different covariates in an adjusted model. The first study compared a 3-compartment model without any covariates to CPB-adjusted models that include factors as sex, weight or premedication as individual covariates. However, these adjusted models did not enhance the prediction of fentanyl plasma concentrations.^31^ In contrast, the 3-compartment model without covariates showed a strong predictive accuracy for fentanyl plasma concentrations during CPB. This finding could provide a scientific foundation for developing dosing regimens of fentanyl during CPB.^30^

### Sufentanil.

Sufentanil showed similar effects to fentanyl regarding its pharmacokinetics and pharmacodynamics. In a 3 compartment model, total sufentanil plasma concentration decreased directly after the onset of CPB, while unbound sufentanil levels and the intercompartmental clearances increased during CPB. After CPB, total sufentanil concentration rose again but did not return to initial prebypass value.^32,33^ Similar to fentanyl, these fluctuations in concentration were considered to be the result of drug redistribution from fat tissue and the restoration of pulmonary blood flow.^32^

A 3 compartment model without covariates was compared with a 3 compartment model either applied with sex or weight as covariate and a CPB-adjusted model. Median prediction error and median absolute prediction error were comparable between all models, favoring the simple 3 compartment model.^32^ Age and body weight as covariates did not improve the predictive ability for changes in sufentanil pharmacokinetics (Supplemental Digital Content, Table 2, http://links.lww.com/AA/F313).^33^

### Remifentanil.

Remifentanil concentrations decrease with the initiation of CPB due to an increase in the volume of distribution, which rises by approximately by 86% during CPB.^27^ However, the effect-site concentration of remifentanil may return to prebypass level depending on patient’s temperature. Elimination clearance of remifentanil decreases with hypothermia, specifically by about 6% for each degree Celsius drop in body temperature.^27^ Consequently, if the infusion rate remains unchanged, total remifentanil concentrations may be higher during CPB.

In cases of moderate hypothermia (27°C) remifentanil plasma concentration can be completely restored to prebypass levels due to decreased clearance, metabolism, and enzyme activity.^27^ Conversely, mild hypothermia (32°C) prolongs the recovery of remifentanil plasma concentration.^27^ Therefore, it is recommended that for moderate hypothermia (27°C), the infusion rate of remifentanil should be reduced immediately after initiation of CPB, while for mild hypothermia infusion rate should be decreased after approximately 20 to 30 minutes.

To maintain consistent blood levels, the infusion of remifentanil should be adjusted to temperature with a suggested reduction of 30% for every 5°C decrease in temperature.^27^ Conversely, in normothermic patients, the increase in volume of distribution during CPB may require higher initial dosing or adjustments in maintenance dosing to achieve the desired analgesic effect.

These findings are also shown in studies focusing on TCI-administered remifentanil.

In the commonly used Minto TCI model actual measured remifentanil plasma concentrations were lower than predicted in the pre- and postbypass period.^28^ Strikingly, this overprediction of the real concentration (median prediction error −26.2% pre-CPB, and −24.9% post-CPB), was not found during CPB. During normothermic CPB, the actual measured remifentanil plasma concentrations were close to those predicted (median prediction error −4.2%, and 0, despite significant hemodilution (20% decrease in hematocrit).^28^ However, during moderate hypothermia, remifentanil plasma concentration was significantly higher than predicted (Table 1). This under-prediction of the real concentration (median prediction error 21.6%) was especially observed during hypothermic episodes, where measured plasma concentrations were over 3 times higher than predicted. This effect was attributed to altered enzymatic hydrolysis activity and therefore reduced clearance.^26,27^

### Alfentanil.

Blake et al^34^ used a 3-compartment model based on lean body mass to calculate alfentanil infusion rates. After CPB initiation until weaning, total alfentanil plasma concentration decreased by 42% without significant alterations in the unbound alfentanil plasma concentration with a calculated constant infusion rate of alfentanil. The most likely explanation is the effect of hemodilution since the ratio of bound- to unbound alfentanil plasma concentration correlated with the plasma concentration of albumin, as well as alpha 1-acid glycoprotein (Supplemental Digital Content, Table 2, http://links.lww.com/AA/F313).^34^ After weaning of CPB and reducing of the alfentanil infusion, both concentrations declined. The authors summarized that alfentanil with a constant infusion rate does not require adjustment for CPB.^34^

## DISCUSSION

This review summarizes the impact of CPB on the pharmacokinetics and -dynamics of commonly used intraoperative continuously administered anesthetics and analgesics in patients undergoing cardiac surgery.

Key findings indicate that CPB significantly alters the pharmacokinetics of propofol, sevoflurane, isoflurane, fentanyl, sufentanil, remifentanil, and alfentanil, resulting in fluctuations in plasma concentrations. Propofol’s plasma concentration decreases due to hemodilution and increased volume of distribution, leading to an increase in the unbound fraction. Therefore, lower doses are required during CPB to achieve the same anesthetic effect. Importantly, only in 2 out of 9 studies with propofol, the anesthetic effect was measured. Additionally, BIS monitoring was used in these studies to measure the anesthetic depth,^16,17^ although evidence from large clinical trials questions its reliability for accurately assessing the depth of anesthesia.^7,39^ Concerning BIS, it is also important to consider that during periods of intense surgical stimulation, hemodynamic instability or the rewarming phase of CPB, BIS monitoring is not validated.

Fentanyl, sufentanil, and alfentanil, show CPB-induced fluctuations in plasma concentration. However, they were less pronounced compared to previous studies reviewed in 2000,^3^ highlighting the relevance of this review in the context of evolving CPB techniques. Finally, although it is clinically important to understand the potential complications resulting from over- or underdosing of anesthetics due to variations in concentration, none of the studies described adverse events as a primary endpoint.

### TCI Models

TCI models for propofol (Schnider, Marsh, and PGIMER) and remifentanil (Minto) were unable to accurately predict the correct plasma concentration during CPB. This inaccuracy may stem from factors such as hemodilution, fluid shifts, temperature chances, but also interaction with other administered anesthetic drugs, particularly opioids, which can alter propofol’s pharmacokinetics.^40^ To address these coadministration effects, the Eleveld model may potentially serve as an alternative approach, as it has demonstrated the ability to predict propofol plasma concentration across a broad patient population undergoing general anesthesia.^41,42^ It simplifies the interaction with opioids by categorizing either being present or not.

However, current literature does not provide a definitive recommendation as to whether anesthesia providers should prefer TCI over non-TCI methods for predicting and dosing propofol plasma concentrations during CPB.

### Influence of the CBP Circuit and Priming

The CPB circuit and priming strategy impact drug pharmacokinetics during cardiac surgery. The extent of hemodilution varies based on the size of CPB circuits, tubing, priming fluid composition, and retrograde autologous priming used. Nevertheless, priming fluid is not discussed within most studies. With a crystalloid-based priming fluid strategy, colloid oncotic pressure including plasma albumin may decrease further compared with a colloidal of albumin-based priming strategy.^43^ Consequently, the unbound fraction of highly protein-bound anesthetics and analgesics might differ between prime fluid strategies, although comprehensive studies on this aspect are currently lacking.

It is important to notice that CPB guidelines do not recommend a sex-based approach to CPB priming.^6^ As practicing perfusionists adhere to these guidelines, it is unlikely that sex-based strategies, such as dilution adjustments based on sex, are routinely used.

### Influence of Patient Factors

Patient factors, particularly sex-related differences, can influence drug pharmacokinetics and pharmacodynamics during CPB. However, it’s crucial to recognize that these differences are not solely determined by biological sex. While female patients generally have higher body fat percentages, lower muscle mass, and smaller blood volumes^44,45^ compared to males, there is considerable individual variation that may overshadow sex-based differences. For instance, a female patient weighing 90 to 100 kg may have a larger estimated blood volume (EBV) than a male patient weighing 50 to 70 kg. This variation in weight and associated EBV can be substantial, potentially exceeding 50% between small men and large women, which is far greater than the purported 20% variation attributed to biological sex alone.

These differences (in small and also obese patients) result in a larger volume of distribution for lipophilic drugs and smaller volume for hydrophilic drugs, leading to lower peak plasma levels for lipophilic drugs and higher peak plasma levels for hydrophilic drugs. These impact the onset and duration of drug action. During CPB, which introduces an additional hydrophilic distribution space, patients’ susceptibility to hemodilution and therefore the potential risk of adverse drug effects may rise.^46^ Normalizing the dose based on weight (mg/kg) can mitigate variations in drug concentration, and selecting the appropriate weight scalar (such as total body weight, lean body weight, fat-free mass, or adjusted body weight) based on body composition and the specific drug may further optimize drug dosing.

Current studies on drug pharmacokinetics during CPB often lack standardization for patient weight and body mass index (BMI). This oversight may lead to misattribution of pharmacokinetic differences to sex when they might be more closely related to individual body composition and size.

Sex differences in drug pharmacokinetics during CPB are primarily related to hormonal factors—particularly in menstruating female patients.^46–50^ Hormonal influences, particularly estrogen levels, can impact drug binding and distribution by potentially reducing the concentration of alpha 1-acid glycoprotein.^51^ Additionally, although, menstrual cycle-related physiological variations in renal function, exceeding 20%, may exist,^47^ the pronounced effects of CPB, such as hemodilution, altered temperature regulation, might overshadow them.

While previous pharmacokinetic models for anesthetics have identified sex as a significant covariate,^41,52,53^ our reviewed studies, specifically focusing on CPB, have not consistently demonstrated this. This discrepancy might be due to the limited representation of female participants (only 30% in some studies),^19,30–32^ and the lack of primary focus on sex as a variable. Moreover, these studies often do not differentiate between pre- and postmenopausal women, despite potential hormonal influences on drug pharmacokinetics.

Further research with weight-matched male and female cohorts is needed to clarify the specific role of sex in pharmacokinetics during CPB.

### Strengths and Limitations

This review has several limitations. The study populations in all included studies were rather small, with only 30% of the participants being female.

Different pharmacokinetic models were investigated and compared, causing heterogeneity, and the effect of concomitant medication on drug concentration was not taken into account.

Some studies used BIS monitoring to stage anesthesia depth. However, these results should be interpreted with caution since BIS monitoring during CPB is not validated and different monitoring systems were used in clinical practice (BIS, Patient State Index (PSI).^9^ Finally, the clinical significance of fluctuations in anesthetic plasma concentrations was not studied.

A strength of this review was its conduction in line with a prospectively designed analysis plan by a multidisciplinary group with experience in cardiothoracic anesthesiology, pharmacology, and cardiothoracic surgery.

### Clinical Recommendations

#### Propofol

Reduce propofol infusion rate: Consider lowering the propofol infusion rate during CPB to avoid excessive plasma levels and minimize hemodynamic and cerebral side effects.

Adjust dosage: After CPB termination, be aware that plasma propofol concentrations may increase, potentially requiring further dose adjustments to maintain appropriate anesthesia depth.

### Sevoflurane/Isoflurane

Adjust dosage: Consider lowering sevoflurane concentration during CPB to account for increased tissue capacity for volatile anesthetics and hypothermia.

Monitor end-tidal concentrations: Use end-tidal sevoflurane measurements as a reliable indicator of plasma concentration to guide anesthesia management.

Avoid high concentrations: Refrain from using high sevoflurane and isoflurane concentrations that induce burst suppression, as this may disrupt CBF autoregulation.

### Fentanyl/Sufentanil

Consider Pre-CPB bolus: Administer an additional bolus before initiating CPB to reduce the initial drop in plasma concentration, while being cautious of potential increases in free drug levels due to hemodilution.

Adjust dosage: Consider reducing the dose if an additional bolus pre-CPB is given, considering the redistribution of opioids and restoration of pulmonary blood flow during CPB to avoid excessive sedation.

### Remifentanil

Infusion rate adjustment: Given the reduced elimination clearance during hypothermia, lower remifentanil infusion rates by 30% for every 5°C decrease in temperature to maintain consistent blood levels during hypothermia.

Dosing considerations: Increased volume of distribution during CPB may require higher initial or adjusted maintenance dosing to achieve effective analgesia in normothermic patients.

### Alfentanil

Considering the decrease in total plasma concentration and the increase in unbound plasma concentration of alfentanil at CPB initiation, it is recommended to maintain constant infusion rates of alfentanil during CPB without adjustment.

### Recommendations for Further Research

Future studies should focus on and prioritize several understudied areas. First, correction for the coadministration of other drugs should be incorporated when analyzing anesthetic plasma concentrations. This adjustment is crucial for better understanding of the specific drug effect being studied. Second, the study population should be larger, sex-balanced, and weight-matched to better understand the clinical significance of sex-related differences, particularly in the context of CPB. One potential approach would be to conduct a comparative analysis of age-matched male and postmenopausal female patients to investigate differences in the pharmacokinetic and -dynamic profiles of anesthetic drugs within the context of CPB. Furthermore, priming, monitoring of anesthesia depth, and concomitant hemodynamic changes should be standardized. The pharmacokinetics and -dynamics of midazolam, a commonly used—and currently underreported—anesthetic in on-pump cardiac surgery, should also be studied. Lastly, the clinical implications of anesthetic use during extracorporeal membrane oxygenation in critically ill should be addressed.

## CONCLUSIONS

Anesthesiologists face complex challenges in managing drug pharmacokinetics during CPB, with current clinical practice focusing on mitigating risks through strategies like processed electroencephalogram monitoring, patient-specific dosage adjustments, and careful anesthetic selection. While adverse events such as intraoperative awareness (0.02%–0.5%) remain relatively rare, they underscore the critical importance of understanding drug behavior during CPB.

Key takeaways include the necessity of considering patient-specific factors, utilizing objective monitoring tools, and recognizing potential drug alterations due to CPB. Significant research gaps remain, particularly in determining optimal anesthetic concentrations, understanding modern CPB technique impacts, exploring sex-specific drug responses, and investigating long-term outcomes.

Future research addressing these knowledge gaps could substantially improve patient safety and outcomes in cardiac surgery, ultimately enabling more precise and personalized anesthetic management strategies during CPB.

## ACKNOWLEDGMENTS

We thank E.M.J.P. Mouws as participating investigator, we thank Faridi Jamaludin for assisting with design and conduct of literature search.

## DISCLOSURES

**Conflicts of Interest:** None. **Funding:** BJA ESAIC Grant 2022 to S. E., J. B., C. v.d.B., J. K., M. H., and H. H. **This manuscript was handled by:** Ken B. Johnson, MD.

## Supplementary Material


