# Supplementary material for: Pharmacokinetics and Pharmacodynamics of Analgesic and Anesthetic Drugs in Patients During Cardiac Surgery With Cardiopulmonary Bypass: A Narrative Review
Source: Anesth Analg. 2025 May 16;142(1):5–14. doi: 10.1213/ANE.0000000000007564 (PMC12677336; doi:10.1213/ANE.0000000000007564)
Supplement: Supplementary file 1 [file ane-142-005-s001.docx]

**Legends**

**Table 1.** Elements of the electronic search

**Table 2.** Characteristics of included studies and main findings on the influence of CPB on the pharmacokinetics of different anesthetic drugs.

**Table 1.** Elements of the electronic search

| **PubMed** |
| --- |
| ((((("Thoracic Surgery"[Mesh] OR "Thoracic Surgical Procedures"[Mesh] OR cardiac surger*[tiab] OR thoracic surger*[tiab] OR cardiothoracic surger*[tiab] OR heart surger*[tiab] OR cardiac surgical procedure*[tiab] OR heart surgical procedure*[tiab]))) AND (("Cardiopulmonary Bypass"[Mesh] OR "Heart-Lung Machine"[Mesh] cardiopulmonary bypass*[tiab] OR heart-lung bypass*[tiab] OR heart-lung machine*[tiab]))) AND (("Pharmacokinetics"[Mesh] OR "pharmacokinetics" [Subheading] OR "pharmacology" [Subheading] OR pharmacodynamic*[tiab] OR pharmaco dynamic*[tiab] OR pharmacokinetic*[tiab] OR pharmaco kinetic*[tiab] OR drug kinetic*[tiab] OR absorption[tiab] OR distribution[tiab] OR elimination[tiab] OR toxicology[tiab] OR mode of action*[tiab] OR mechanism of action*[tiab] OR pharmacologic action*[tiab]))) AND (("Anesthesia, Cardiac Procedures"[Mesh] OR "Anesthetics"[Mesh] OR "Anesthetics, Combined" [Pharmacological Action] OR "Anesthetics, Intravenous" [Pharmacological Action] OR "Anesthetics, Inhalation" [Pharmacological Action] OR "Anesthetics, Local" [Pharmacological Action] OR "Anesthetics, Dissociative" [Pharmacological Action] OR cardiac anaesthesia*[tiab] OR cardiothoracic anaesthesia*[tiab] OR anesthetic agent*[tiab] OR anesthetic drug*[tiab] OR anesthetic effect*[tiab] OR propofol*[tiab] OR ketamine*[tiab] OR sevoflurane*[tiab] OR isoflurane*[tiab] OR opiates*[tiab] OR fentanyl*[tiab] OR sufentanil*[tiab] OR morphine*[tiab] OR remifentanil*[tiab] OR alfentanil*[tiab] OR midazolam*[tiab])) |
| **Embase** |
| ('thoracic surgery'/exp OR 'thoracic surgical procedure' OR 'cardiac surgery':ti,ab OR 'thoracic surgery':ti,ab OR 'cardiothoracic surgery':ti,ab OR 'heart surgery':ti,ab OR 'cardiac surgical procedure':ti,ab OR 'heart surgical procedure':ti,ab) AND ('cardiopulmonary bypass'/exp OR 'heart-lung machine'/exp OR 'cardiopulmonary bypass':ti,ab OR 'heart-lung bypass':ti,ab OR 'heart-lung machine':ti,ab) AND ('pharmacokinetics'/exp OR 'pharmacology'/exp OR 'pharmacodynamics':ti,ab OR 'pharmaco dynamics':ti,ab OR 'pharmacokinetics':ti,ab OR 'pharmaco kinetics':ti,ab OR 'drug kinetics':ti,ab OR 'absorption':ti,ab OR 'distribution':ti,ab OR 'elimination':ti,ab OR 'toxicology':ti,ab OR 'mode of action':ti,ab OR 'mechanism of action':ti,ab OR 'pharmacologic action':ti,ab) AND ('cardiac anesthesia'/exp OR 'anesthetics'/exp OR 'anesthetics, combined'/exp OR 'anesthetics, intravenous'/exp OR 'anesthetics, inhalation'/exp OR 'anesthetics, local'/exp OR 'anesthetics, dissociative'/exp OR 'cardiac anaesthesia':ti,ab OR 'cardiothoracic anaesthesia':ti,ab OR 'anesthetic agent':ti,ab OR 'anesthetic drug':ti,ab OR 'anesthetic effect':ti,ab OR 'propofol':ti,ab OR 'ketamine':ti,ab OR 'sevoflurane':ti,ab OR 'isoflurane':ti,ab OR 'opiates':ti,ab OR 'fentanyl':ti,ab OR 'sufentanil':ti,ab OR 'morphine':ti,ab OR 'remifentanil':ti,ab OR 'alfentanil':ti,ab OR 'midazolam':ti,ab) |

**Table 2.** Characteristics of included studies and main findings on the influence of CPB on the pharmacokinetics of different anesthetic drugs

| **Author, year** | **Study design and population** | **N** | **Anesthetic drug** | **Main findings and clinical implication** |
| --- | --- | --- | --- | --- |
| Mathew et al.(1), 2016 | Randomized CABG, Valve, | 23 | Propofol | Plasma concentration decreased during CPB. Plasma concentration increased after CPB, comparable with pre-CPB. Clinical implication: risk of underdosing during CPB, risk of overdosing after CPB. |
| Barbosa et al.(2), 2009 | Non-randomized, on-pump vs. off pump CABG | 20 | Propofol | Plasma concentration increased during CPB compared with off-pump surgery. Longer time to awakening after infusion stop, despite shorter biological half-life values. Higher total plasma clearance. Increased bispectral index after CPB. Clinical implication: risk of overdosing after CPB. |
| Silva-Filho et al.(3), 2018 | Non-randomized, on-pump vs. off pump CABG | 19 | Propofol | Two to five-fold increase in unbound propofol plasma concentration compared with off pump surgery. Time to extubation longer after CPB due to higher volume of distribution and longer distribution half-life. Increased plasma clearance. Clinical implication: risk of overdosing during CPB. |
| Takizawa et al.(4), 2006 | Randomized, on-pump cardiac surgery | 30 | Propofol | Two fold increase in unbound protocol plasma concentration during CPB. Total propofol plasma concentration not different. BSR increased during CPB in patients receiving 6- compared with 4 mg kg^-1^ h^-1^. Clinical implication: risk of overdosing during CPB. |
| Yoshitani et al.(5), 2003 | Randomized, CABG, Valve | 45 | Propofol | CPB reduced plasma propofol concentrations. Gradually increase in BSR during CPB in patients receiving 5- or 6 mg kg^-1^ h^-1^, not in patients receiving 4 mg kg^-1^ h^-1^. Clinical implication: risk of underdosing during CPB, risk of overdosing after CPB. |
| Hiraoka et al. (6), 2004 | Not specified | 19 | Propofol | CPB reduced total plasma propofol concentration, while unbound plasma propofol concentration increased by 2-fold. Plasma propofol concentration gradually increased to pre-bypass values. After completion of CPB, unbound plasma propofol concentration gradually fell but did not return to the initial pre-CPB value.  Mean hepatic extraction ratio for propofol exceeded 0.8 and remained constant throughout surgery. No differences in total body clearance of propofol were found before, during and after CPB.  Total protein, albumin and alpha1-acid glycoprotein plasma concentrations decreased significantly during CPB. By contrast, free fatty acids concentrations increased during CPB, and returned to baseline on termination of CPB.  Clinical implication: risk of overdosing during and after CPB. |
| Meroni et al.(7), 2016 | Observational, mitral valve | 10 | Sevoflurane | End tidal sevoflurane plasma concentration decreased at initiation of CPB and gradually increase during CPB. Overall sevoflurane plasma concentration comparable. Clinical implication: risk of underdosing during CPB, risk of overdosing after CPB. |
| Nitzschke et al.(8), 2013 | Observational, CABG, Valve,  CABG/Valve,  LV aneurysm  repair | 30 | Sevoflurane | Sevoflurane plasma concentration decreased directly after initiation of CPB and gradually increased during CPB, but remained lower during and after CPB compared to pre-bypass sevoflurane plasma concentration. BIS did not differ. Clinical implication: risk of underdosing during CPB. |
| Freiermuth et al. ,(9) 2016 | Observational, CABG during mini-CPB | 31 | Sevoflurane and Isoflurane | Sevoflurane and isoflurane used only during CPB. Wash-in and wash-out kinetics of sevoflurane and isoflurane were similar using a mini-CPB system, despite the differences in relative blood-gas solutbilities of isoflurane (1.38) versus sevoflurane (0.66’, from which one should expect a faster wash-in and -out kinetics with sevoflurane. Temp (34 degrees of Celsius) resulting in increase in BG solubility, hemodilution decrease. Higher dose of sevoflurane was needed to reach a similar depth of anesthesia (BIS 30-45) |
| Hudson et al.(10), 2003 | Non-randomized, CABG | 90 | Fentanyl | Small fluctuations (25%) in plasma concentration measured at onset and separation from CPB. Fat tissue storage and storage of pulmonary blood during CPB buffers rise and fall of plasma concentration. Clinical implication: Risk of underdosing during CPB, risk of overdosing after CPB. |
| Hudson et al.(11), 2004 | Observational, CABG | 21 | Sufentanil | Decrease (26%) in total plasma concentration at onset of CPB and transient increase (47%) after separation from CPB. Equal plasma concentration at the end of surgery compared with before separation from CPB. Clinical implication: Risk of overdosing after CPB. |
| Jeleazcov et al.(12), 2012 | Randomized, CABG | 38 | Sufentanil | Total plasma concentration decreased during CPB, while unbound plasma concentration and free sufentanil fraction in plasma increased. Elimination and intercompartmental clearances increased during CPB. This study only included male patients. Clinical implication: none. |
| Michelsen et al.(13), 2001 | Randomized, CABG | 68 | Remifentanil | Volume of distribution increased (86%) with CPB initiation in a two-compartment model resulting in decreased remifentanil plasma concentration. Rapid recovery during hypothermia due to decreased elimination clearance. Clinical implication: risk of underdosing during normothermic CPB. |
| Blake et al.(14), 2003 | Non-randomized, CABG | 25 | Alfentanil | Total plasma concentration decreased. Unbound plasma concentration was not altered significantly from sternotomy until CPB release. Ratio bound to unbound plasma concentrations during CPB correlated with plasma concentrations of albumin and alpha 1-acid glycoprotein (hemodilution). Clinical implication: none. |

Abbreviations: BIS, bispectral index; BSR, burst suppression rate; CPB, cardiopulmonary bypass. a: Group 6 is defined as 6 mg kg^-1^ h^-1^, b: Group 4 is defined as 4 mg kg^-1^ h^-1^.

**References**

1. Mathew PJ, Sailam S, Sivasailam R, Thingnum SK, Puri GD. Performance of target-controlled infusion of propofol using two different pharmacokinetic models in open heart surgery - a randomised controlled study. Perfusion. 2016;31(1):45-53.

2. Barbosa RA, Santos SR, White PF, Pereira VA, Silva Filho CR, Malbouisson LM, Carmona MJ. Effects of cardiopulmonary bypass on propofol pharmacokinetics and bispectral index during coronary surgery. Clinics (Sao Paulo). 2009;64(3):215-21.

3. Silva-Filho CR, Barbosa RAG, Silva CV, Jr., Malbouisson LMS, Carmona MJC, Jorge-Santos SRC. Application of a pharmacokinetics-pharmacodynamics approach to the free propofol plasma levels during coronary artery bypass grafting surgery with hypothermic cardiopulmonary bypass. Clinics (Sao Paulo). 2018;73:e178.

4. Takizawa E, Hiraoka H, Takizawa D, Goto F. Changes in the effect of propofol in response to altered plasma protein binding during normothermic cardiopulmonary bypass. Br J Anaesth. 2006;96(2):179-85.

5. Yoshitani K, Kawaguchi M, Takahashi M, Kitaguchi K, Furuya H. Plasma propofol concentration and EEG burst suppression ratio during normothermic cardiopulmonary bypass. Br J Anaesth. 2003;90(2):122-6.

6. Hiraoka H, Yamamoto K, Okano N, Morita T, Goto F, Horiuchi R. Changes in drug plasma concentrations of an extensively bound and highly extracted drug, propofol, in response to altered plasma binding. Clin Pharmacol Ther. 2004;75(4):324-30.

7. Meroni R, Gianni S, Guarnieri M, Saglietti F, Gemma M, Zangrillo A, Bignami E. Feasibility of Anesthesia Maintenance With Sevoflurane During Cardiopulmonary Bypass: A Pilot Pharmacokinetics Study. J Cardiothorac Vasc Anesth. 2017;31(4):1210-7.

8. Nitzschke R, Wilgusch J, Kersten JF, Trepte CJ, Haas SA, Reuter DA, et al. Changes in sevoflurane plasma concentration with delivery through the oxygenator during on-pump cardiac surgery. Br J Anaesth. 2013;110(6):957-65.

9. Freiermuth D, Mets B, Bolliger D, Reuthebuch O, Doebele T, Scholz M, et al. Sevoflurane and Isoflurane-Pharmacokinetics, Hemodynamic Stability, and Cardioprotective Effects During Cardiopulmonary Bypass. J Cardiothorac Vasc Anesth. 2016;30(6):1494-501.

10. Hudson RJ, Thomson IR, Jassal R, Peterson DJ, Brown AD, Freedman JI. Cardiopulmonary bypass has minimal effects on the pharmacokinetics of fentanyl in adults. Anesthesiology. 2003;99(4):847-54.

11. Hudson RJ, Thomson IR, Jassal R. Effects of cardiopulmonary bypass on sufentanil pharmacokinetics in patients undergoing coronary artery bypass surgery. Anesthesiology. 2004;101(4):862-71.

12. Jeleazcov C, Saari TI, Ihmsen H, Schuttler J, Fechner J. Changes in total and unbound concentrations of sufentanil during target controlled infusion for cardiac surgery with cardiopulmonary bypass. Br J Anaesth. 2012;109(5):698-706.

13. Michelsen LG, Holford NH, Lu W, Hoke JF, Hug CC, Bailey JM. The pharmacokinetics of remifentanil in patients undergoing coronary artery bypass grafting with cardiopulmonary bypass. Anesth Analg. 2001;93(5):1100-5.

14. Blake DW, Royse CF, Royse AG, Bjorksten AR, Soeding PF, Pang J. Alfentanil infusion as a component of intravenous anaesthesia for coronary artery bypass surgery with "fast-track" recovery. Anaesth Intensive Care. 2003;31(2):181-3.
